# Expression profiling of *prospero *in the *Drosophila *larval chemosensory organ: Between growth and outgrowth

**DOI:** 10.1186/1471-2164-11-47

**Published:** 2010-01-19

**Authors:** Laure Guenin, Mahatsangy Raharijaona, Rémi Houlgatte, Fawzia Baba-Aissa

**Affiliations:** 1 Institut Pasteur, Pathogénomique Mycobactérienne Intégrée, 25, Rue du Dr. Roux, 75724 Paris Cedex 15, France; 2 Université de Bourgogne, Facultés des Sciences, Unité Mixte de Recherche 5548 Associée au Centre National de la Recherche Scientifique, 6, Bd Gabriel, 21 000 Dijon, France; 3 INSERM, U915, Nantes, F-44000, France; 4 Université de Nantes, l'Institut du Thorax, Nantes, F-44000, France

## Abstract

**Background:**

The antenno-maxilary complex (AMC) forms the chemosensory system of the *Drosophila *larva and is involved in gustatory and olfactory perception. We have previously shown that a mutant allele of the homeodomain transcription factor Prospero (*prosVoila1*, *V1*), presents several developmental defects including abnormal growth and altered taste responses. In addition, many neural tracts connecting the AMC to the central nervous system (CNS) were affected. Our earlier reports on larval AMC did not argue in favour of a role of *pros *in cell fate decision, but strongly suggested that *pros *could be involved in the control of other aspect of neuronal development. In order to identify these functions, we used microarray analysis of larval AMC and CNS tissue isolated from the wild type, and three other previously characterised *prospero *alleles, including the *V1 *mutant, considered as a null allele for the AMC.

**Results:**

A total of 17 samples were first analysed with hierarchical clustering. To determine those genes affected by loss of *pros *function, we calculated a discriminating score reflecting the differential expression between *V1 *mutant and other *pros *alleles. We identified a total of 64 genes in the AMC. Additional manual annotation using all the computed information on the attributed role of these genes in the *Drosophila *larvae nervous system, enabled us to identify one functional category of potential Prospero target genes known to be involved in neurite outgrowth, synaptic transmission and more specifically in neuronal connectivity remodelling. The second category of genes found to be differentially expressed between the null mutant AMC and the other alleles concerned the development of the sensory organs and more particularly the larval olfactory system. Surprisingly, a third category emerged from our analyses and suggests an association of *pros *with the genes that regulate autophagy, growth and insulin pathways. Interestingly, EGFR and Notch pathways were represented in all of these three functional categories. We now propose that Pros could perform all of these different functions through the modulation of these two antagonistic and synergic pathways.

**Conclusions:**

The current data contribute to the clarification of the *prospero *function in the larval AMC and show that *pros *regulates different function in larvae as compared to those controlled by this gene in embryos. In the future, the possible mechanism by which Pros could achieve its function in the AMC will be explored in detail.

## Background

In *Drosophila*, some external sensory organs found in the anterior region of larvae are composed of many neurons and support cells that seem to represent an aggregation of several sensory units. This is the case for the antenno-maxillary complex (AMC) that forms the chemosensory system of the *Drosophila *larva. The chemosensory apparatus of the larval head is formed during embryogenesis [1] and consists essentially of three major sensilla complexes on the cephalic lobe, the dorsal (DO), terminal (TO) and ventral organs (VO), and a series of pharyngeal sensilla [2,3]. While the DO appears to be a mixed smell and taste organ, the TO, VO and pharyngeal sensilla may be exclusively gustatory [4-7].

In previous studies, we described a mutant allele of the transcription factor *prospero *(*Voila1, V1*) that is associated with several alterations in both AMC and the CNS [8]. *V1 *homozygotes die before forming pupae. Surviving larvae remain much smaller than wild-type individuals and are impaired for their response to salt and sucrose [9]. Using a set of previously characterised *Voila *alleles (*prosV*) that express different levels of Prospero (Pros) protein, we found that the level of Pros expression detected in the embryonic precursor region of the AMC, was related to the degree of alteration of larval taste [10]. In embryonic and larval AMC, Pros is expressed in the same cell cluster (~50 cells), including neuronal cells (~10 cells) and many accessory cells but no glial cells [8].

*The pros *gene encodes a transcription factor protein that contains a highly divergent putative homeodomain and a conserved Prospero domain that are both necessary for sequence-specific DNA binding and Prospero nuclear localisation [11-13]. Pros is known to be expressed in neuronal precursor cells [14,15] and participates in cell fate decision in both neuroblasts and sensory organ lineages [16-19]. Pros has been shown to control axonal and dendritic outgrowth [15], glial development [20,21] and to be a key regulator of mitotic activity in embryos [22]. Pros affects several cell cycle genes and can either promote or inhibit them depending on the cellular or the developmental context [23,24]. More recently Choksi et al. [25] showed that in the embryonic nerve cord Pros repressed target genes such as cell cycle genes required for self-renewal, and was also required to activate genes involved in terminal differentiation.

In a previous study on the larval antenno-maxillary complex, we showed that loss of *pros *function did not alter the mitotic activity or the final number of neurons. By contrast, many neural tracts connecting the AMC to the CNS are affected [8]. Therefore, it is possible that one key role of Pros in the larval AMC is to control the expression of genes involved in neuron-specific development such as axon routing and/or neurite outgrowth. However, as Pros is expressed in non-neuronal cells in the AMC (accessory cells), it is likely that, it regulates genes that are also involved in other functions.

In order to identify the Pros target genes associated with this organ, we performed microarray analysis on larval AMC tissue isolated from the wild type, the *V1 *mutant and two previously characterised *prospero *alleles [8], *V13 (prosVoila13) *and *V24 *(*prosVoila24*, see also Table 1). To establish the AMC specificity of these genes, we included analysis of samples from isolated larval CNS for these four alleles.

**Table 1:** Overview of the phenotypes associated with the different *prosV *alleles.

Allele	Genotype	Stage of lethality	Larval taste response	Pros expression in AMC	Pros expression in CNS	Axonal routing in AMC
***V14***	Wild type (complete *PGal4 *remobilization)	Viable	Normal	Normal	Normal	Normal

***V13***	Partial *pGal4 *excision (remaining of 718 bp)	Young adult < 2 days old	Normal	Normal	altered	Normal

***V24***	Partial *pGal4 *excision (remaining of 7400 pb)	pupal	Intermediate	Normal	altered	Normal

***V1***	full length *pGal4 *transposon (12900 pb)	larva	Altered	absent	altered	Misrouting

**(redrawn from Guenin et al**. [8]**)**.

In the *prosV1 *(*V1*) allele, the full length *PGal4 *transposon is inserted upstream of the *pros *coding region (-216 bp). *prosV14 *(*V14*) results from the correct and total remobilization of the transposon, in this strain the wild type phenotype is restored. In *prosV24 *(*V24*) and *prosV13 *(*V13*), the *PGal4 *element has been partially removed, respectively 7400 and 718 bp remain inserted 216 bp upstream the *pros *start site. The peak of developmental lethality, the taste response of late homozygous 2nd instar larva, Pros expression level in larvae and axonal misrouting are indicated for each *prosV *allele.

The larval taste response was measured towards 0.1 *M *sucrose and 0.3 *M *NaCl concentration that are known to respectively attract or repulse wild type *Drosophila*. *V1 *mutants were indifferent to both substances (altered taste response), *V24 *showed an intermediate response: they were repulsed by NaCl but remained indifferent to sucrose and *V13 *and *V14 *present a normal taste response to both substances. The Pros expression pattern is indicated by comparison to the *V14 *wild type: In the AMC, *V1 *showed no Pros expression but for the other alleles, Pros expression pattern was similar to *V14*. In the CNS, all mutant alleles showed a distinct altered expression pattern as compared to the wild type (further descriptions of the Pros pattern are found in the text and in [Additional file 2]).

Our findings indicate that, in this sensory organ, *pros *is mainly associated with the regulation of genes that are essential for correct routing of neural processes and synaptic transmission. Many of these genes are involved in the development and remodelling of the nervous system during metamorphosis. Interestingly, we also found that loss of *pros *function induced the misregulation of a subset of genes important for growth, and autophagy. Finally, the possible role of *EGFR *(the epidermal growth factor receptor) and the *N *(Notch) pathway in regulating all of these functions is discussed.

## Results

### The developing AMC and Pros expression

**Figure 1:**
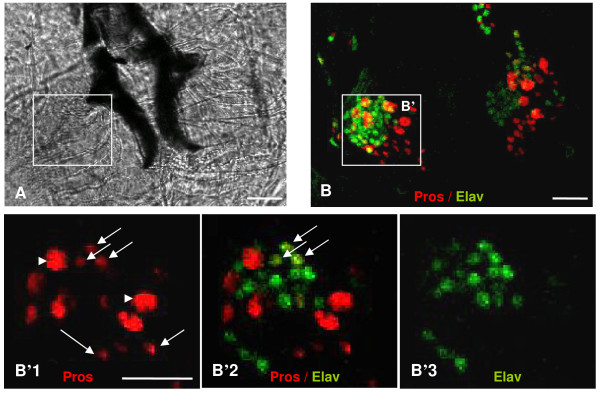
**AMC region from third instar larvae observed by optical microscopy**. (A) Bright-field view of the larval AMC region (dorsal view, anterior down), the hooks appear in dark. Cells that constitute the AMC are located on either side of the hooks. (B) 3D reconstruction of AMC (TO +DO), labelled with Pros (red) and Elav (green) that labels neuronal cells. (B'1-3) Zoomed view of a confocal section of the framed region in B showing respectively the Pros (B'1), Pros/Elav (B'2) and Elav (B'3) staining; Anti-Prospero labels two types of Pros expressing cells (Pros+): large (arrowheads in B'1) and small cells (arrows in B'1). Some of the small Pros+ cells express Elav (B'2). Scale bars represent 10 μm.

We have previously shown [8] that in the developing AMC, Pros is always expressed in the same cluster of cells. In addition, neither mitotic activity nor apoptosis was observed during the third instar larval stage or at late embryonic stages suggesting that the final number of Pros expressing cells is fixed before the end of embryogenesis [8]. This hypothesis was further confirmed by analysing mitotic activity in the developing wild type AMC (*prosVoila14, V14) *using an H3p marker. Our results showed that the H3p labelling disappeared completely after the embryonic stages 12-13 [Additional file 1] indicating that additional cells are not provided until the last larval stage. However, some Pros expressing cells grew in size at the LIII stage. Scoring the different Pros+ cell type morphology (Figure 1, Table 2), we found that the wild type larval AMC (TO and DO) was composed of 8 (± 1) large Pros+ cells (most probably accessory cells) and 40 (± 4.1) small Pros+ cells. Among the latter, 10.7 (± 2.8) were neuronal cells [8]. Pros is never expressed in glial cells. Interestingly, *pros *loss of function affected the axonal pathway in the embryonic AMC, but produced the correct number of neuronal cells [8] and curiously induced an excess of glial cells, which, we suspect, originated from incorrect peripheral glial cells migration. Therefore, if *Pros *was expressed in the same number of cells in the embryo and larvae and since no additional cell division was seen after stage 13, it is likely that *pros *is not involved in cell fate choice in the larval AMC.

**Table 2:** Pros expressing cells in the larval AMC

Cell types	Large cells	Small cells		
**Alleles**	**Pros+**	**Pros+**	**Pros+/Elav+**	**Elav+**

**V14**	8 ± 1	40.9 ± 4.1	10.7 ± 2.8	65.8 ± 1.4

**V1**	0 ***	0 ***	0 ***	62.3 ± 0.9

We have quantified the number of Pros expressing cells (Pros+) and neuronal cells (Elav+) in the third instar larval AMC of wild type (*V14*) and *V1 *mutants. We distinguish two types of Pros+ cells on the basis of their size: large and small cells. Some small Pros+ cells express Elav markers and are probably differentiated neurons. In *V1 *mutants, no more Pros protein is detected in the larval AMC, but the number of neurons remains unchanged.

To better clarify the role of *pros *in the AMC, we carried out microarray analysis on wild type (*V14*) and three *prospero *mutants *(V1, V13, V24, see *also Table 1), which present different expression levels of Pros [8,10]. *V1 *is considered as a null *pros *allele for the larval AMC as no Pros protein is detected in this organ. It presents an abnormal taste response to sucrose and NaCl (indifferent to both substances), and shows an alteration of the neural connections between the AMC and CNS as well as arborisation defects in larval neuromuscular junction [10]. In the *V1 *larval CNS, Pros is still expressed but at a lower level than in the wild type *V14*. *V1 *larval CNS also shows several defects, which include early initiation of cell death and abnormal sub-cellular localization of the Pros protein [Additional file 2: supplemental figure A].

*V13 *and *V24 *are both derived from the *V1 *allele and result from incomplete excision of the *PGal4 *transposon, respectively 7400 and 718 bp remain inserted upstream of the *pros *start site (see also Table 1). A previous study showed that *V13 *[8] and *V24 *(Personal communication) present a correct structure of the larval AMC and a normal expression level of *pros *mRNA. No variation was observed in the number of glia or neuron cells and the pattern of Pros expression was similar to that of the wild type *V14*. Since, *V14, V24 *and *V13 *present the same expression pattern in the AMC and have the same genetic background, individual variations (independent of Pros expression), can be more easily eliminated by the use of several but similar fly lines.

In the CNS both alleles overexpress (at different levels) the *pros *mRNA but present a distinct pattern as regards to the mitotic activity or to the Pros and Elav (neuronal cells marker) labelling [Additional file 2]. In the ventral nerve cord *V13 *showed a clear hyperplasia due to an excess of neurons [Additional file 2: supplemental figure B]. Finally, mitotic activity, revealed by anti-pHistone-H3 antibody (H3p) [Additional file 2: supplemental figure C] was strongly increased, especially in the Optic Lobes of *V24*. In conclusion, each of *V24 *and *V13 *allele presented a distinct abnormal pattern as compared to the wild type *V14*. Therefore, we used in the CNS *V13 *allele only as its Pros expression pattern was previously published.

All these alleles were used to for the transcriptome analysis since they have the same genetic background.

Most specifically, to identify the genes that are misregulated in the AMC, the comparison was made between the null AMC *V1 *mutant and the other alleles *V14, V24, V13 *as all three present the same wild type expression pattern in this structure.

It should be mentioned that the role of Pros in the larvae CNS was not investigated in this study. The CNS was used to be compared with AMC (for this only *V14, V1 *and *V13 *alleles were selected) and to determine whether the putative candidate genes identified in the AMC could be found in the CNS. For this latest purpose, we decided to limit the microarray analysis in the CNS to the comparison between *V14 *and *V1*, since the *V13 *allele presents an intermediate Pros expression pattern in the CNS. This avoids misinterpretation of the CNS data.

### Transcription profile of *prosV1 *larvae

Expression data of a total of 17 samples were analysed, including both CNS and AMC samples for the four *prosV *alleles and 2 to 3 independent RNA extractions for each allele. We searched for sets of genes participating in the same biological function (with correlated expression) and differentially expressed between *prosV *mutants. We used the Discriminating Score (DS, see also Methods section) as the detection method for differential expression, smoothed on the hierarchical clustering tree to detect peaks of correlated genes. This method had the advantage of detecting peaks of optimal size. This size could not be known *a priori*. A DS score can be assigned to each node in the dendrogram. The node corresponding to the maximum DS score was chosen as the node best fitting the peak.

**Figure 2:**
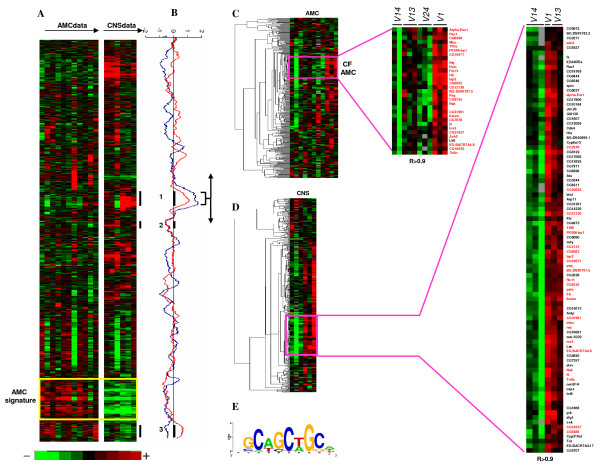
**Gene expression analyses**. (A) Hierarchical clustering of 5950 genes for a total of 17 samples relative to the larval AMC and CNS of the different *prosV *mutants. Each row represents a gene and each column a sample. For each organ, the samples at the top of the image are classified according to the severity of their phenotypes (from wild type to the most severe phenotype: *V14, V13, V24 *and *V1*). Each cell in the matrix corresponds to the expression level of one gene in a sample (see colour scale at the bottom of the image). The yellow frames represent the AMC tissue specific signature and contain the genes that are differentially expressed between AMC and the CNS independently of Pros expression. (B) Discriminating scores (DS) smoothed in a window of 100 genes, calculated between *V1 *and other *prosV *in the AMC (in red), and between *V1 *and *V14 *in the CNS (in blue), among the gene clusters. In the AMC, three peaks, annotated 1, 2 and 3 (black bars), appear to be enriched in differentially expressed genes, they have been associated respectively to "cell fate commitment", "proteasome complex" and "signal transduction" ontologies. (C) Hierarchical clustering of the 306 genes present in peak 1 in the AMC. Pink frame zooms on a set of highly correlated (r>0.9) genes that are differentially expressed between *V1 *and all other alleles in the AMC. These genes are referenced on the right according to the *Drosophila *nomenclature (see also Table 3). The dendrogram on the left represents correlation distances between the profiles of the studied genes. Differentially expressed genes indicated in red were common with CNS. (D) Same as (C) in CNS samples. The Pink framed region contains 86 genes which are referenced on the right according to the *Drosophila *nomenclature. Differentially expressed genes indicated in red were common with AMC. (E) Motif found in the promoting region of the 28 genes common to AMC and CNS (genes indicated in red).

The results were displayed using TreeView [26]. As it can be seen in the Figure 2A, a cluster of genes differentially expressed in AMC and CNS tissues and in a Pros independent manner was observed. The genes of this cluster (AMC tissue specific signature) were clearly overexpressed in the AMC while the same genes were underexpressed for all alleles in the CNS (Figure 2A).

**Table 3:** Genes identified as putative Pros targets and their manual annotation

Genes	symbol	Biological function in larvae (manual annotation)
**Cell fate commitment (GO:0045165, p = 0.0003)**		

αEst1*	CG1031	Sensory neuron morphogenesis

Art3*	CG6563	Not studied in larvae

Ash2*	CG6677	Neurite outgrowth, synapse formation, growth, sensory organ development

CG10632*	CG10632	Unknown

CG10671*	CG10671	Unknown

CG3021*	CG3021	Unknown

CG31637*	CG31637	Unknown

CG31961*	CG31961	Unknown

CG31731*	CG31731	Unknown

***CG6388***	CG6388	Neurite outgrowth

CG7878*	CG7878	Unknown

CG8155*	CG8155	Unknown

DPAL1*	CG12130	Neuropeptide biosynthesis

FK506-bp1*	CG6226	Autophagy; growth

Ftz-F1*	CG4059	Autophagy, sensory organ formation, olfaction

Hb*	CG9786	Labial segment formation including sense organ

Iap2*	CG8293	Autophagy, sensory organ development

Inx3*	CG1448	Not studied in larvae

Keren*	CG32179	Autophagy, sensory organ development

Mbo*	CG6819	Tracheal system development

Nak*	CG10637	Not studied in larvae

Nej *	CG15319	Synaptic transmission, autophagy

Notch*	CG3936	Neurite outgrowth, nutrient sensing/growth, sense organ formation, olfaction

Pelo*	CG3959	Not studied in larvae

Psq*	CG2368	Sensory organ development, olfaction

Rac1*	CG2248	Neurite outgrowth, sensory organ development

Tollo*	CG6890	Synaptogenesis, wing development, immune response

TFIIs*	CG3710	Not studied in larvae

*LK6*	CG17342	Autophagy growth, nutrient sensor mechanism,

**Proteasome complex (GO:0000502, p < 10^-5^,)**		

Pros 26.4	CG5289	Neuronal remodelling, Autophagy

*Prosβ2*	CG3329	Neuronal remodelling, Synaptic transmission, autophagy, sensory organ formation

*Pros26*	CG4097	Neuronal remodelling, Synaptic transmission, autophagy

*Prosα6*	CG18495	Neuronal remodelling, Synaptic transmission, autophagy

*Prosα7*	CG1519	Neuronal remodelling, Synaptic transmission, autophagy

*ProsMA5*	CG10938	Not studied in larvae

*RPN1*	CG7762	Neuronal remodelling, Autophagy

*RPN2*	CG11888	Neuronal remodelling, Autophagy

*RPN5*	CG1100	Neuronal remodelling, Autophagy

**Signal transduction (GO:0004871, p = 0.0008)**		

*Bnl*	CG4608	Neurite outgrowth

*CaMKI*	CG1495	Synaptic transmission

*CG10011*	CG10011	Unknown

*CG10702*	CG10702	Autophagy

*CG1088*	CG10882	Unknown

*CG31714*	CG31714	Unknown

*CG4839*	CG4839	Unknown

*CG5790*	CG5790	Unknown

*CG7536*	CG7536	Unknown

*CG7800*	CG7800	Unknown

*CKII α*	CG17520	Sensory organ development

*Dok*	CG2079	Sensory organ development

*EGFR*	CG10079	Neurite outgrowth, synapse formation, growth, autophagy sensory organ development, olfaction

*feo*	CG11207	Mitotic spindle organisation

*Gek*	CG4012	Actin polymerisation

*Gwl*	CG7719	Neurite outgrowth, synaptic transmission, mitotic cell cycle

*InaC*	CG6518	Not studied in larvae

*Kdelr*	CG5183	Not studied in larvae

*LimK1*	CG1848	Neurite outgrowth, synaptic transmission

*Lok*	CG10895	Cell cycle, DNA damage checkpoint

*Loco*	CG5248	Not studied in larvae

*Mcr*	CG7586	Olfaction

*PhKγ*	CG1830	Not studied in larvae

*Pvr*	CG8222	Hemocyte formation, dorsal closure, macrochaete formation

*Rh7*	CG5638	Not studied in larvae

*Toll-6*	CG7250	Not studied in larvae

The 64 genes found highly correlated in the peak 1, 2 and 3 are grouped according to their respective GO annotation class. The most significant classes of genes enriched in our list are "Cell fate commitment", "proteasome complex" and "signal transduction". The p value indicates the probability for a given ontology to be associated at random to this cluster. The first 28 genes indicated in bold and by an asterisk share a common DNA motif (**CAGCTG**) in their promoter and were also found to be differentially expressed between *V1 *and *V14 CNS*.

The last column on the right specifies the known biological function (manual annotation) of these genes in the *Drosophila *larvae (Full references can be found in the main text and in [Additional file 5: Supplemental Tables S2-S4]). This manual annotation allowed the attribution of biological function to 37 genes. Some genes have either never been studied in larvae or their respective functions are currently unknown. By contrast to the GO annotation (mostly deduced from embryos), the use of manual annotation indicates that the dysfunction of *pros *leads in larvae to the misregulation of genes that mostly deal with neurite outgrowth, growth and autophagy and sensory organ formation (mainly olfactory). All genes were found to be overexpressed in the mutant *V1 *AMC except for the 9 genes associated with the Proteasome complex GO annotation.

In the next step, we determined those genes affected by loss of *pros *function for each organ with the DS. For the AMC, as *V13, V14 *and *V24 *have a normal Pros expression pattern in this structure, the DS was calculated for each gene between *V1 *and all other alleles. For the CNS, since *V13 *exhibited a distinct pattern [Additional file 2], the DS for each gene was calculated between *V1 *and the *V14 *alleles only.

To visualize groups of correlated genes that were differentially expressed between *V1 *and other alleles, the DS score obtained for the AMC or CNS was plotted alongside the hierarchical clustering and smoothed in a sliding windows of 100 genes. As shown in Figure 2B, different peaks can be detected. Each peak represents co-expressed genes sensitive to Pros expression. To avoid the analysis of non-significant variations, we decided to assess the biological functions of these groups of genes. We therefore searched for significant enrichments of Gene Ontology terms (GO) in each cluster using GoMiner [27]. In the AMC, only 3 peaks (peaks 1-3) could be associated with significant GO functions (Figure 2B peaks 1, 2 and 3). Complete gene lists for peaks 1-3 are presented in [Additional file 3 and 4].

In peak 3, the 26 genes overexpressed in *V1 *AMC were significantly associated with the over-represented GO term « signal transducer activity » (GO:0004871, p = 0.0008, see also Table 3). Significant enrichments of the GO term "proteasome complex" (GO:0000502, p < 10^-5^, see also Table 3) were found for 9 genes in peak 2. All of these genes were underexpressed in *V1 *AMC. Peak 1, with the highest DS, was common to AMC and CNS and associated with the over-represented GO term "cell fate commitment" (GO:0045165, p = 0.0003). Inside this peak, a cluster of 29 genes overexpressed in *V1 *AMC and highly correlated (r>0.9, Figure 2C, Table 3) was isolated and we found a cluster of 86 genes overexpressed in *V1 *CNS and correlated (r>0.9, Figure 2D, see also [Additional file 5: Supplemental Table S1]) in the same peak. Interestingly, among the 86 CNS genes, 28 (represented in red in the Figure 2D) also belonged to the AMC gene list (see also Table 3 and [Additional file 5: Supplemental Table S1]).

Finally, to assess our microarray analysis, we quantified the expression of seven selected genes by Q-PCR. As shown in Table 4, our results were consistent with the microarray data except for the *hb (hunchback) *gene found to be overexpressed in the CNS but not in the AMC. Because the two methods have different sensitivities, the magnitude of the change determined by microarray and real time PCR is not the same. The orientation of changes, however, is identical.

**Table 4:** Validation of microarray data using real time PCR.

	Relative expression level *V1*/*V14*				
	
		AMC		CNS	
**Gene Primers**		**Microarray**	**Q-PCR**	**Microarray**	**Q-PCR**

*caps*	F 5'GCAGCCTGGATGAAGGTTTA 3' R 5'ATGGCGCAGCCATAGTAGTC 3'	1.38	0.63	**3.8**	**2.38**

*Cdk4*	F 5' TACAACAGCACCGTGGACAT 3' R 5' GGTCCAGCTGATTCTTTTCG 3'	0.95	1.3	**4.99**	**2.5**

*hb*	F 5' CCTTCCAGTGCGACAAATG 3' R 5' ATCCGCACAACGGTACTGA 3'	**6.71**	**0.85**	**6.38**	**1.6**

*Iap2*	F 5'AAGGACTGGCCGAATCCCAACATC 3' R 5' CGTTGCACCAAACACACTTC 3'	**3.69**	**2.16**	**6.48**	**1.9**

*nak*	F 5'AGGAAGCATCACAGCAAAAT 3' R 5'GCACCAGGAGCAGCTGTAAC 3'	**1.75**	**1.36**	**0.97**	**1.95**

*nej*	F 5'AATGGATCCAACGGATATCTCT 3' R 5'CTGATCCGACCAGCCACTAT 3'	**3.26**	**1.63**	**3.79**	**3.75**

*Notch*	F 5'AACACCGTTCGCGGAACTGATACCG 3' R 5'GGTTTTGCCATTGAGTTGTG 3'	**2.9**	**1.76**	**8.96**	**2.52**

The relative expression level (*V1*/*V14*) of selected genes was measured using the Q- PCR or microarray analysis data; Our results were consistent with the microarray data except for the *hb (hunchback) *gene found to be overexpressed in the CNS but not in the AMC. The values in gray correspond to the genes found differentially expressed between *V1 *and *V14 *in the CNS but not in the AMC using microarray analysis. Accordingly, no significant variation was found for these genes in the AMC, using Q-PCR.

An interesting observation was that we found highly correlated genes responding to Pros variation in both CNS and AMC. This tight correlation in two different tissues could suggest that these genes may be controlled by common transcription factors including Pros. We searched for transcription factor binding sites that were common to these 28 genes. We used a Gibbs sampling method [28] on the -1700 to +300 bp promoter region of these genes. This method allowed the determination of degenerated motifs, described by a position weight matrix (PWM), in a set of sequences by iterative sampling.

We found a motif shown as a Logo [29] in Figure 2E. It notably included a CAGCTG core. This motif shows weak and probably not significant similarities with other Pros motifs previously proposed for *Drosophila*: TAAGNCT [25], CACNNCT [12], TAAGACG [30]. Therefore, additional experiments are now necessary to see whether the motif identified in this study could really bind the Prospero transcription factor *in vivo*.

### Additional manual annotation to specify the role of *pros *putative target genes in *Drosophila *larvae

Our microarray analyses showed that peak 1 contained 29 overexpressed genes associated with the GO annotation, "cell fate commitment" (Table 3). These data are not consistent with our previous studies showing that *pros *is not involved in cell fate determination in the larval AMC [8]. We were intrigued by this discrepancy and therefore we looked more deeply for the function of these genes in the larvae. Interestingly, though most of the genes present in this peak were associated with cell fate determination in embryos, such evidence was mostly missing for the larvae peripheral nervous system (PNS). Thus, it is likely that the GO annotation was mostly deduced from the reported function of these genes in *Drosophila *embryos. Therefore, to more specifically identify the role of these genes in the *Drosophila *larva PNS, we used additional manual annotation.

The first step consisted in compiling all of the information available on the role attributed to each of the 64 genes identified from peaks 1-3, but most specifically in *Drosophila *larvae. The information was collected using Flybase, mutant analysis, associated phenotypes, research articles and microarray data. As much as possible, we selected only data that reported the function of these genes in the larval nervous system and more specifically the sensory system. Out of the 64 genes, we found that 27 had unknown biological functions or had not been documented in larvae (Table 3). Since the genes encoding the subunit of the proteasome complex are under-expressed and the remaining genes are overexpressed in the *V1 *AMC, a description of the phenotype generated by the upregulation and downregulation of the corresponding genes in larvae was given [Additional file 5: Supplemental Tables S2-S4]. Though many phenotypes were available for a gene, we selected only those reported for the nervous system and preferentially for the PNS.

**Figure 3:**
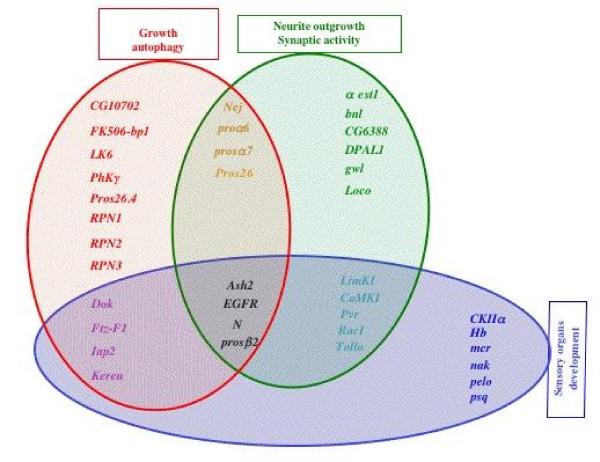
**Schematic representation of the overlapping function attributed to the AMC putative Pros target genes**. The functional categories were established using a manual annotation (the criteria used for this annotation are indicated in the text, see also for further phenotypic description and corresponding references [Additional file 5: Supplemental Tables S2-S4]). The three functional groups identified are represented by three distinct colored sets. The genes located at the intersection between two sets can assume both functions. It should be noted that the genes indicated in black (*EGFR, Notch, Ash2 and prosβ*2) belong to the three functional groups: neurite outgrowth, sensory organ development, and growth/autophagy.

Analysis of the resulting compiled data on larva [Additional file 5: Supplemental Tables S2-S4] revealed that the 37 genes fell into at least one of the following functional categories: (1) neurite outgrowth and/or synaptic transmission; (2) growth, autophagy; (3) sensory organ (mainly olfactory) development. A list of these genes and their associated annotation terms is summarized in Table 3, and a schematic representation is given in Figure 3. As shown in Figure 3, some genes can be associated with two functional classes, and four genes (*EGFR, Notch, Ash2 *and *prosβ*) are associated with the three functional categories: neurite outgrowth, autophagy, and olfactory system development.

### Genes involved in neural processes and synaptic transmission are misregulated in the *V1 *AMC mutant

One of the functional categories deduced from manual annotation associates some putative Pros target genes with neurite outgrowth and/or synaptic transmission (Figure 3). Although synaptic transmission and neurite outgrowth belong to different functional categories, we decided to keep these genes in the same class since many of them are involved in both synaptic transmission and neurite outgrowth.

Mostly, the genes are overexpressed in the larval *V1 *AMC, (Table 3). It has been reported that the upregulation of most of these candidates inhibits neurite outgrowth in larval neurons (reported phenotypes are shown in [Additional file 5: Supplemental Table S2]). This is clearly the case for *EGFR (Epidermal Growth Factor Receptor), N (Notch), bnl (branchless) *and *Rac1 *[31-33] whose overexpression was previously shown to inhibit axon extension in larval neurons. This is also the case for *gwl (greatwall*), *limK1 (lim-kinase 1)*, *Nej (Nejire) *or *CG6388, Pvr*, whose upregulation induces axon pathfinding defects or impaired neurotransmitter release in the larval neuromuscular junction (NJM) [34-38]. Interestingly, similar axon pathfinding and NMJ defects were observed in the *V1 *mutant [8].

We have noticed that most of the genes included in this functional class can also drive neural connectivity remodelling in larvae, a process particularly important during metamorphosis. *EGFR, Notch*, *bnl, Rac1 *[31-33], and the genes associated with the ubiquitin-proteasome system were all reported to be involved in axon extension/retraction, pruning and morphogenesis of larval peripheral sensory neurons [Additional file 5: Supplemental Table S2]. Indeed, though most larval sensory neurons will degenerate during metamorphosis, some persist as neurons and undergo stereotyped pruning of their dendrites and axon terminal branches during early metamorphosis [39].

We were not surprised to find that the genes encoding the different proteasome subunits (*Prosα7, Prosβ2, Prosα6, Prosα26) *are downregulated in *V1 *AMC (Figure 3, Table 3). Indeed, the acute regulation of their protein level is a primary determinant of protein turnover and neurotransmission strength [40,41].

Recently, an elegant study of Choksi et al. [25] showed that *pros *is required for activation of neuronal differentiation genes in embryos and identified *N, bnl, LimKI, EGFR and PVR, prosα6 *as putative *pros *targets in embryos. This reinforces our finding suggesting that in the larval AMC, *pros *plays a crucial role in the modulation of neuronal activity through the control of genes involved in neurotransmission and synaptic plasticity.

### Loss of *pros *function alters the expression of genes involved in autophagy and growth

The second functional group that emerges from our analysis includes candidates that play a critical role in the control of autophagy, a process used to provide energy and nutrients during metamorphosis and early adulthood.

The association between Pros and the regulation of autophagy is mainly attested by the upregulation of genes such as *CG10702, EGFR, Keren *(EGFR ligand), *Ftz-F1 (Ftz transcription factor 1), FK506-bp1 (FK506-binding protein 1), Iap2 (Inhibitor of apoptosis 2), nej, Notch *and genes associated with proteasome complex (Table 3, Figure 3, see also [Additional file 5: Supplemental Table S3]).

Some of the genes cited above were also found to mediate cell growth (Table 3 and [Additional file 5: Supplemental Table S3]). However, it is not yet clear if the overexpression of these genes systematically inhibits cell growth. For example, the upregulation of *LK6 (protein serine/threonine kinase) *or *FK506*-bp1 leads to either the activation or inhibition of cell growth in a context-dependent way [42-44].

We have already mentioned that Notch and EGFR pathways were involved in neurite outgrowth (see above), interestingly, we found that these two pathways were also associated with both the regulation of autophagy and cell growth control [45,46] (Figure 3), suggesting that Pros could mediate all of these functions through the modulation of these two pathways.

Our finding that *pros *is associated with the expression of genes involved in growth and or autophagy is consistent with the phenotypic defects observed in *V1 *homozygote mutants: i) individuals died before reaching puparium formation; ii) surviving larvae and pupae remained much smaller than wild-type individuals; iii) numerous labelled cells were observed in the fat body using PGal4 enhancer trap line *V1 *[9].

It is interesting to note that many of the genes found in this functional group are directly or indirectly associated with insulin-signalling pathways and more specifically the insulin/TOR (target of rapamycin) pathway, an important mediator of growth, autophagy and nutrient sensing [Additional file 5: Supplemental Table S3].

### Pros and the olfactory system

Pros was detected both in the terminal (TO: mainly gustatory) and in the dorsal (DO: mainly olfactory) organs of the larval AMC [8]. Accordingly, we found that *pros *loss of function in the AMC induced the upregulation of all candidate genes (except *prosβ2*) that were known to be involved in the development of sensory organs (Figure 3, Table 3). Most of our knowledge on the function of these genes came from studies done on adult *Drosophila *sensory organs [Additional file 5: Supplemental Table S4]. For example, it has been reported that mutations in the genes *ash2 or ckII alpha (Casein kinase II alpha subunit) *can elicit supernumerary or ectopic adult sensory organs [47,48]. Similarly, overexpression of *Iap2 or limK1 *induces respectively additional macrochaetes [49] or ectopic glomeruli in adult antennae lobes [36]. The transmembrane receptor *Notch *and the epidermal growth factor receptor *EGFR *also seem to play an important role in the organisation, remodelling and function of the olfactory system [Additional file 5: Supplemental Table S4]. This confirms previous observations which showed that they were respectively required for selecting the sensory organ precursor lineages [50,51] and for the development of some of the neurons and cuticular structures of the antenno-maxillary sensory complex [52].

## Discussion

### Pros may regulate genes essential for neurite outgrowth and remodelling

In the AMC, the transcription factor Prospero is expressed in a cluster of cells (composed of neuronal and support cells, but not glial cells) that emerge during embryonic life and are maintained till the end of the larval stages. In embryos, Pros was reported to be involved in cell fate decision and in cell-cycle control. By contrast, our earlier data from the larval AMC rather suggested that *pros *could assume more restricted functions, such as the control of neuron-specific functions [8]. The present study confirms this hypothesis and shows that in the chemosensory organs dedicated to larval olfactory and gustatory sensing, *prospero *could regulate genes involved in neurite outgrowth and synaptic transmission.

Since *pros *was clearly shown to control axonal and dendritic outgrowth [53], we cannot exclude the possibility that the connection of *pros *with several genes that drive synaptic activity could be the indirect consequence of its involvement in neurite outgrowth control. In this respect, it is interesting to mention that a recent study [54] showed that axon targeting of the R7 *Drosophila *photoreceptor cells to their synaptic partner requires R7-specific transcription factor Prospero. These authors proposed that Pros could promote cell-type-specific expression of sensory receptors and cell-surface proteins regulating synaptic target specificity.

As previously mentioned, some of the genes identified in this functional class are also involved in neural connectivity remodelling. How can this be achieved if the AMC is completely histolysed? In fact, in *Drosophila*, not all sensory neurons degenerate; Some larval neurons persist and remodel to take on a new role in the adult system [55,56]. During the metamorphosis larval arbors of these neurons are pruned back and new adult-specific arbors are generated through a subsequent period of outgrowth. It seems that the neurites of these persistent larval neurons are used to partly guide axons of adult sensory neurons towards and within the CNS [55]. Therefore, histolysis and remodelling are two processes that are achieved during metamorphosis and could concern distinct neurons.

Does Pros play any role in AMC neuronal remodelling? We cannot actually answer the question. However, it has been previously reported that the insulin and epidermal growth factor signalling pathways, as well as ubiquitin-specific proteases are all required for the regulation of *Drosophila *neuronal remodelling [57]. Interestingly, all of these components emerge clearly from our analysis.

Actually, no work was done on the *Drosophila *larvae anterior sense organ in order to check whether some of the sensory neurons (which have also an embryonic origin) persist and remodel to take place in the adult olfactory or gustatory system. Therefore, the question is left open. At least the answers will provide important insights into the mechanisms that govern developmental plasticity in insect nervous systems.

In summary, our data collected from larval AMC and the previous genome wide expression profiling done on embryos [25] confirms that *pros *is associated with the regulation of neuronal specific genes. In this respect, it is essential to note that except for a few genes (126), most of the Pros target genes identified (~1000) in Choksi et al. [25] were not represented on our microarrays. For this reason, and because our experiments were performed on isolated individual larval tissues, it is not possible to determine whether the genes identified by these authors are specifically expressed in embryos and/or in tissues other than AMC.

### Prospero and the insulin pathway

In *Drosophila*, the insulin/TOR signalling pathway [58] is divided into two branches. The insulin and its downstream effectors P13 and FOXO (forkhead box) represent one branch [59] of this pathway, while the other branch acts through the TOR family of Serine-Threonin kinases [60,61]. It has been shown that the insulin/TOR signalling pathway inhibits autophagy (For review see [58]) and controls growth by regulating ribosome biogenesis and protein biosynthetic capacity [62,63]. Columbani et al. [63] demonstrated that the TOR pathway is a nutritional checkpoint that participates in the systemic control of larval growth emanating from the Fat body.

Our microarray analysis has revealed a group of highly correlated *pros *candidate genes (correlation index: 0.9) that are either controlled by the insulin/TOR signalling pathway or are directly involved in the signalling cascade. This is the case for *Ash2 *[64] which was found to be regulated by TOR signalling. Similarly, FK506-bp1 affects autophagy through the modulation of FOXO [44] and *Lk6 *was reported to be a direct FOXO Target [62]. Therefore it seems that in the larval AMC, Pros could be associated with growth, autophagy and nutrient sensing through the regulation of genes that are directly or indirectly linked to the insulin/TOR pathway. Interestingly, *TOR *was found to be differentially expressed in the *V1 pros *mutant in the CNS [Additional file 5: Supplemental Table S1].

## Conclusion

As previously described, loss of *pros *function in the AMC induced several alterations including axon pathfinding defects and abnormal growth and taste responses. This is consistent with our microarray results showing that in the larval AMC, Pros expression is associated with the regulation of genes involved in the control of neurite outgrowth, mediation of growth and autophagy and in the organisation and function of the olfactory system. The mechanism by which all of these functions are achieved by *pros *in the AMC is presently not known but EGFR and/or Notch pathways could play a central role. Several lines of evidence are in favour of this hypothesis.

1- Four ligands are known to bind EGFR receptor: Keren, Gurken, Spitz, and Vein [65]. Two of these were identified as potential targets of Prospero: Keren in both larval AMC and CNS and Gurken (Grk) in the larval CNS only (see Table 3 and [Additional file 5: Supplemental Table S1]). Moreover, Notch and EGFR were identified as putative Pros target genes in both embryos [25] and larval AMC, indicating that they could play a central role.

2- It has been reported that EGFR signalling is required for the development of some of the neurons and cuticular structures present in the AMC [66,67]. In this respect, it is interesting to point out that EGFR involvement has been reported during the development of mouse gustatory epithelia in the palate and tongue [68].

3- The expression of Notch, EGFR and Pros have been shown to be tightly linked. It has been demonstrated that normal levels of Pros expression in photoreceptor R7 cells in the *Drosophila *eye require EGFR signalling as well as Notch activation [69,70]. In addition, a recent analysis has shown that in R7 cells, Notch and EGFR cooperate in a complex way to promote *pros *transcription [71].

Although these data suggest that Notch and EGFR could play a central role in the mechanism by which Prospero carries out its function in the larval AMC, this hypothesis has still to be validated. In the future, it will be of great interest to explore in detail the mechanism by which all of these functions are accomplished by the homeodomain transcription factor Prospero.

## Methods

### Drosophila strains

All strains were maintained on standard cornmeal and yeast medium at 25°C. The *prosV *strains used in this study have already been described [72]. Briefly, *prosV13 (V13) *and *prosV24 *(*V24) *derived from the same *prosV1 (V1) *allele which contains the full length *PGal4 *transposon inserted upstream (-216 bp) of the *pros *coding region. The resulting behavioural and developmental anomalies observed in these mutants have been previously reported [8], and are summarised in Table 1. Additional descriptions of the expression pattern of these different alleles in the larval CNS are also provided in the [Additional file 2].

### Isolation of AMC and CNS tissue

Around 150 larvae were used to obtain the AMC and CNS samples. The anterior region of the Larva was dissected to isolate CNS and AMC. The AMC region is not a well-defined tissue but is rather constituted by a small group of cells located in front of the hooks. Therefore, to maintain AMC integrity we kept the cuticle around it as well as the hooks.

### Immunohistochemistry experiments

Staining experiments were performed as previously described by Guenin et al. [8] Briefly, isolated larval AMC from embryos at stage 10-17 were incubated with various primary antibodies: MR1A mouse anti-Prospero at 1:4 dilution, rat anti-Elav at 1/1000 (a neuronal marker; provided by A. Giangrande), and rabbit anti-phosphohistone H3 at 1/1000 (a marker for mitotic activity; SIGMA). The following secondary antibodies were used to visualize these primary antibodies: anti-mouse Cy3 at 1/100 (Sigma); anti-mouse Alexa 594 anti-rat Alexa 488 at 1/400 (Molecular probes, USA); anti-rabbit Alexa 488 at 1/400 (Molecular probes, USA). AMC and CNS were mounted on Vectashield (Vector Laboratories, CA) before inspection under a fluorescence microscope (Leica DMRB) or a confocal microscope (Leica 4SD).

### RNA extraction and cDNA labelling

Total RNA from third instar larvae was extracted from isolated AMC and CNS, according to Guenin et al. [8]. Four independent extractions were performed for each sample condition. RNA integrity was checked on denaturing formaldehyde agarose gels. The presence of clear bands corresponding to the 28s and 18s RNA with a 2:1 ratio and the absence of a smear were used to assess the RNA quality. Total RNA (3.0 μg) were treated with RQ1 RNase-free DNase (Promega) and reverse transcripted in presence of ^33^[P] dATP (Amersham Pharmacia Biotech, Bucks, United Kingdom), Random Primers and the reverse transcriptase (Maloney Murine Leukaemia Virus, Invitrogen).

### Microarray experiments

Nylon membrane microarrays provided by the TAGC platform (Marseille-Nice Genopole) were used. They contained 7500 amplification PCR products of unique full length cDNA clones from the *Drosophila *Gene collection release version 1.0 (Berkeley *Drosophila *Genome project). To verify the quality of spotting on the microarrays and the amount of DNA accessible for each spot, a vector probe (labelled oligonucleotide common to all spotted PCR products) hybridization was performed. Hybridization of ^33^[P] labelled probes was conducted for 24 h at 68°C in 500 μl of hybridization buffer (5× SSC, 5× Denhardt's, 0,5% SDS). After 3 washing of 1 h in 500 ml of washing buffer (0,1× SSC, 0,2% SDS) at 68°C, arrays were exposed overnight to phosphor imaging plates which were scanned using a BAS 5000 (Fuji, Raytest, Paris France).

### Data processing and analysis

Signal intensities were quantified using ArrayGauge software (V1.3; Fuji, Paris, France). All images were carefully inspected to exclude spots with overestimated intensities due to neighbourhood effects. Artefacts were eliminated by visual inspection. Spots were excluded from the quantification, if they were contaminated by overflowing neighbouring spots or if artefacts are present on the membrane. Overflowing spots were also eliminated. One sample was discarded (CNS *V14) *because of bad vector signals. The variability due to experimental conditions was eliminated by using a local weighted scattered plot smoother analysis (LOWESS, [73]). The data were then filtered and only values found to be twice the mean local background value were kept. Correlation coefficients between expression measurements of two identical alleles ranged from 0.74 to 0.93 for the same tissue. Data were then log transformed.

Genes belonging to the same biological function or cell type are known to exhibit correlated expression [74]. Thus we searched for sets of genes with correlated expression that were differentially expressed between mutants. We used a method to detect groups of correlated genes and a statistical method to detect differential expression among these groups. Different methods were able to detect correlated expressions. We chose hierarchical clustering which has the advantage of not fixing a priori the number of classes or the number of genes per class. The statistical method used to detect genes differentially expressed between the wild type and *pros *mutants was the Discriminating Score [75], which is very similar to the widely used SAM method [76]. As the expected number of genes was not known, we calculated an average DS in a sliding window on the cluster. This allowed us to detect peaks of the optimal size. These peaks corresponded to genes with correlated expression and differentially expressed between mutants. For the clustering, we used the Cluster program with Pearson correlation distance and average linkage as the aggregation strategy. The results were displayed using TreeView [26]. The DS measures the difference in gene expression between 2 groups of samples. If M1 represents mean expression of a given gene in wild type samples, and M2 the mean expression of the same gene in *pros *mutant samples, and SD the standard deviation of this gene in all considered samples, DS = (M1-M2)/(SD). As *V13, V14 *and *V24 *have a normal Pros expression pattern in the AMC, they were considered wild type. The DS between *V1 *and all of the other alleles for the AMC was calculated for each gene. As *V13 *and *V24 *exhibited a distinct pattern in the CNS, for each gene, we calculated a DS between the *V1 *and *V14 *allele for the CNS. The score for each gene was then smoothed by calculating the mean score in a sliding window of 100 genes.

Finally, to highlight very tightly correlated genes in the main peak (peak 1) for each tissue, we performed a second hierarchical clustering with the genes of this peak, separately in the CNS and AMC.

The complete dataset is available through the National Center for Biotechnology Information (NCBI), in the Gene Expression Omnibus database http://www.ncbi.nlm.nih.gov/geo/ under the GSE12178 accession number.

### Functional Annotation

Functional annotations of gene clusters were performed using GoMiner software [27] and the Gene Ontology database [77]. GoMiner determines significant enrichments of GO terms in a cluster of genes. This is performed by comparing the frequencies of each GO term in the cluster and in the microarray using Fisher's exact test.

### Research of a putative common motif in the promoter of co-expressed genes

Promoting regions of co-expressed genes were collected from the Ensembl ftp site http://www.ensembl.org/index.html The sequence located from -1700 to +300 bp according to the +1 transcription start site of each gene was extracted. Interspersed and simple repeats were masked. These sequences were searched for a common motif using the Gibbs sampling method [28] available at the RSA Tools website: http://rsat.ulb.ac.be/rsat/. This method allowed to determine degenerated motifs described by a position weight matrix (PWM, a probabilistic model of residue frequencies at each position), in a set of sequences by iterative sampling. The initialisation step of the algorithm selects a random subsequence in each sequence to be searched. The predictive step builds a PWM from all the subsequences except one. The sampling step selects a new subsequence from the excluded sequence using a weighting strategy based on the PWM scores. The predictive and the sampling steps are iterated a given number of times or until convergence. We performed many Gibbs sampling runs as this method is stochastic. The motif found was identified in most of the runs. We used a Logo representation [29] to show the information content of the PWM.

### Q-PCR validation

In order to validate the Microarray results, seven genes were selected and their expression levels were quantified by Q-PCR for both *prosV1 *and *prosV14*. For each RNA extraction used for microarrays, a sample was collected in order to perform Q-PCR experiments. Reverse transcriptions were done from 2 μg of total RNA as described in Guenin et al. [8]. The selected genes and the corresponding primers, designed using Primer Express™ (Applied Biosystems) parameters, are indicated in Table 4. Q-PCR reactions were performed with 1:10 diluted cDNA in 2× SybrGreen PCR master mix (Applied Biosystems) with each specific primer (See table 4) or control primers (*actin 5C *F 5'GCCCATCTACGAGGGTTATGC3' and *actin 5C *R 5'CAAATCGCGACCAGCCAG3'). Signals were measured with ABI Prism 7000™ Sequence Detection System software (Applied Biosystems). All signal thresholds to be compared were standardized with the *actin 5C *mRNA [8].

## Authors' contributions

LG and FB generated the microarray data and drafted the manuscript. LG carry out immunohistochemistry and Q-PCR experiments. LG, MR and RH, performed the biostatistic analysis. MR performed the motif discovery. FB generated the *Drosophila *larvae data compilation for manual annotation. FB and RH provided direction and oversight of the experiments. FB holds the grant. All authors read, corrected and approved the final manuscript.

## Supplementary Material

Additional file 1 **Mitotic Activity in the AMC region, in stage 11 and stage 16 embryos**. Wild type embryos from stage 11 to 16 were stained with Pros (red) and H3p (green) which label cells in division. (A) Mitotic activity is observed until stage 11-12 (arrow) while no more activity is detected from stage 16 embryos (B) in the AMC. Scale bar correspond to 20 μm. Click here for file

Additional file 2 **Expression pattern of *V24 *in the third instar larval CNS**. The Pros (A) and Elav (B) expression pattern as well as the mitotic activity (C) of *V24 *are shown as compared to the previously reported pattern of *V14, V13 *and *V1 *alleles [8]. Although *V24 *and *V13 *present the same expression pattern in the AMC, the situation is different in the CNS. As it can be seen Pros (A) and Elav labeling (B) are distinctive for both alleles in the region delimiting the two hemispheres and the Optic lobes (OLs). As compared to *V13*, *V24 *presents a decrease of the staining in this region for these two markers (B). In ventral nerve cord (VNC), *V13 *shows an important hyperplasia (arrowheads) due to an excess of neurons. In *V24*, the VNC extremity presents a bifida aspect (arrow). The mitotic activity, revealed by anti-pHistone-H3 (H3p) (C) is strongly increased in *V24*, especially in the OLs. Click here for file

Additional file 3 **List of the AMC genes represented in the 3 peaks obtained after the application of the discriminating score between *V1 *and all other alleles for the AMC samples**. **Peak 1**: Differentially expressed genes involved in cell fate commitment. **Peak 2**: Differentially expressed genes involved in the proteasome complex. **Peak 3**: Differentially expressed genes involved in signal transducer activity. Click here for file

Additional file 4 **List of the CNS genes represented in the peak obtained after the application of the discriminating score between *V1 *and *V1 *4 for the CNS samples**. The peak contains differentially expressed genes involved in cell fate commitment. Click here for file

Additional file 5 **Supplemental Table S1: Genes found to be differentially expressed between *V1 *and *V14 *CNS**. We found 86 genes that are highly correlated (coeff> 0,9). Among the 86 genes, 28 were also found to be overexpressed in the *V1 *AMC (column on the left, gene names are indicated in bold) and contain the common putative *pros *DNA motif in their promoter. The 58 genes present in the two columns on the right are specifically overexpressed in *V1 *CNS as compared to *V14 *CNS. **Supplemental Table S2: Phenotypic data related to the candidate genes involved in neurite outgrowth and/or synaptic transmission**. The criteria used for the description of the phenotypes were as follows: (1) if many larval phenotypes were available for a gene, we selected only those observed in the nervous system and preferentially in the peripheral nervous system (PNS). (2) If no larval phenotype was available for a gene, we selected those observed in the embryonic and/or adult PNS. (3) If available, the effect of the upregulation or downregulation of these genes is mentioned respectively for those that are overexpressed or underexpressed in *V1 *AMC. (4) All studies mentioned were done in *Drosophila melanogaster*. **Supplemental Table S3: Phenotype data related to the candidate genes involved in growth and autophagy**. The criteria used for the description of the phenotypes were the same as those for Table S2. **Supplemental Table S4: Phenotype data related to the candidate genes involved in sensory organ development and most particularly in olfaction (in bold)**. The criteria used for the description of the phenotypes were the same as those for Table S2. Click here for file
