# Supplementary figures and images for: Expression profiling of prospero in the Drosophila larval chemosensory organ: Between growth and outgrowth
Source: BMC Genomics. 2010 Jan 19;11:47. doi: 10.1186/1471-2164-11-47 (PMC2826315; doi:10.1186/1471-2164-11-47)

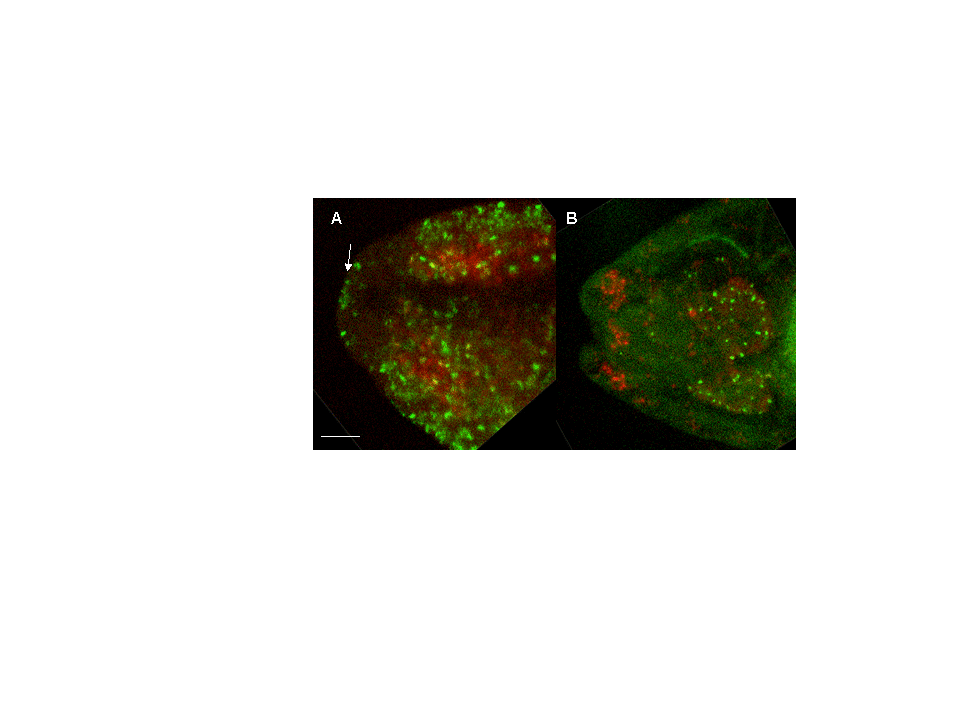

Supplement: Additional file 1 — Mitotic Activity in the AMC region, in stage 11 and stage 16 embryos. Wild type embryos from stage 11 to 16 were stained with Pros (red) and H3p (green) which label cells in division. (A) Mitotic activity is observed until stage 11-12 (arrow) while no more activity is detected from stage 16 embryos (B) in the AMC. Scale bar correspond to 20 μm. [file 1471-2164-11-47-S1.GIF]

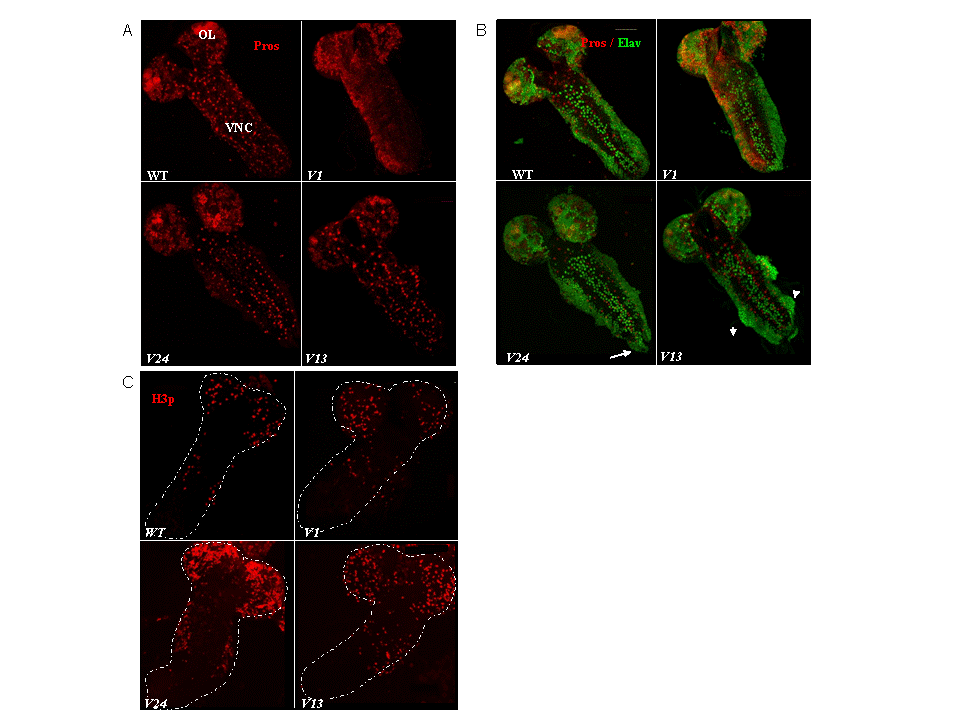

Supplement: Additional file 2 — Expression pattern of V24 in the third instar larval CNS. The Pros (A) and Elav (B) expression pattern as well as the mitotic activity (C) of V24 are shown as compared to the previously reported pattern of V14, V13 and V1 alleles [8]. Although V24 and V13 present the same expression pattern in the AMC, the situation is different in the CNS. As it can be seen Pros (A) and Elav labeling (B) are distinctive for both alleles in the region delimiting the two hemispheres and the Optic lobes (OLs). As compared to V13, V24 presents a decrease of the staining in this region for these two markers (B). In ventral nerve cord (VNC), V13 shows an important hyperplasia (arrowheads) due to an excess of neurons. In V24, the VNC extremity presents a bifida aspect (arrow). The mitotic activity, revealed by anti-pHistone-H3 (H3p) (C) is strongly increased in V24, especially in the OLs. [file 1471-2164-11-47-S2.GIF]
