# Supplementary material for: Expression profiling of prospero in the Drosophila larval chemosensory organ: Between growth and outgrowth
Source: BMC Genomics. 2010 Jan 19;11:47. doi: 10.1186/1471-2164-11-47 (PMC2826315; doi:10.1186/1471-2164-11-47)
Supplement: Additional file 5 — Supplemental Table S1: Genes found to be differentially expressed between V1 and V14 CNS. We found 86 genes that are highly correlated (coeff> 0,9). Among the 86 genes, 28 were also found to be overexpressed in the V1 AMC (column on the left, gene names are indicated in bold) and contain the common putative pros DNA motif in their promoter. The 58 genes present in the two columns on the right are specifically overexpressed in V1 CNS as compared to V14 CNS. Supplemental Table S2: Phenotypic data related to the candidate genes involved in neurite outgrowth and/or synaptic transmission. The criteria used for the description of the phenotypes were as follows: (1) if many larval phenotypes were available for a gene, we selected only those observed in the nervous system and preferentially in the peripheral nervous system (PNS). (2) If no larval phenotype was available for a gene, we selected those observed in the embryonic and/or adult PNS. (3) If available, the effect of the upregulation or downregulation of these genes is mentioned respectively for those that are overexpressed or underexpressed in V1 AMC. (4) All studies mentioned were done in Drosophila melanogaster. Supplemental Table S3: Phenotype data related to the candidate genes involved in growth and autophagy. The criteria used for the description of the phenotypes were the same as those for Table S2. Supplemental Table S4: Phenotype data related to the candidate genes involved in sensory organ development and most particularly in olfaction (in bold). The criteria used for the description of the phenotypes were the same as those for Table S2. [file 1471-2164-11-47-S5.PDF]

Additional file 5

Table of content

Table S1: Genes found to be differentially expressed between *V1* and *V14* CNS.

Table S2: Phenotype data related to the candidate genes involved in neurite outgrowth and/or synaptic transmission.

Table S3: Phenotype data related to the candidate genes involved in growth and autophagy.

Table S4: Phenotype data related to the candidate genes involved in sensory organ development and most particularly in olfaction (in bold) .

Table S1: Genes found to be differentially expressed between *V1* and *V14* CNS.

Name	Biological or Cellular Function	Name	Biological or Cellular Function	Name	Biological or Cellular Function
αEst1	Hydrolase activity	*Antp*	transcription factor activity	*CG5037*	Integral membrane protein
Ash2	Transcription regulator activity	*Arp11*	Protein binding	*CG5080*	
Art3	Protein amino acid methylation	*Atu*	-	*CG5850*	
CG6388	tRNA processing	*Bap55*	Structural constituant of cytoskeleton	*CG6296*	Phopholipase A1 activity
CG7878	Nucleic acid binding	*blot*	Neurotransmitter transport	*CG6311*	
CG31731	transport	*caps*	Axon guidance	*CG7357*	Nucleic acid binding
CG8155	Small GTPase regulator	*Cdk4*	Cell cycle	*CG7971*	RNA splicing
CG31961	Protein binding	*cenB1A*	Regulation of GTPase	*CG8129*	Protein binding
CG31637	Sulfotransferase activity	*CG10184*	Amino acide metabolism	*CG8444*	Asymmetric protein localisation
CG3021	tRNA processing	*CG10681*	Protein binding	*CG9972*	
CG10632	Protein binding	*CG12009*	Chitin binding	*Cyp310a1*	Oxidoreductase activity
CG10671	unknown	*CG14230*	Nucleic acid binding	*Cyp6a13*	Oxidoreductase activity
DPAL1	Protein modification process	*CG1515*	Vesicul mediated transport	*dlg1*	
Ftz-F1	Ligand-dependant nuclear receptor	*CG15835*	Cell communication	*dyl*	Srucrtural constituant of cuticule
FK506-bp1	Protein folding	*CG1607*	transport	*elav*	
Hb	Transcription activator activity	*CG17068*	Protein binding	*fz*	Asymmetric protein localisation
Iap2	Protein binding	*CG17806*	Protein binding	*GM130*	Golgi organisation and biogenesis
Inx3	Gap junction channel activity	*CG1868*		*grk*	Cell fate commitment
Keren	MAPKKK cascade	*CG18769*		*Hrs*	Neurotransmitter secretion
Mbo	Protein nucleus import	*CG31301*	Nucleic acid binding	*Int6*	Translation initiation factor
Nak	Serine/threonine kinase	*CG3238*		*JhI-26*	Nucleic acid binding
Nej	Transcription coactivator	*CG3271*		*Klc*	Microtubule motor activity
Notch	Protein binding,	*CG3344*	Proteolysis and peptidolysis	*l(2)44DEa*	Fatty acid metabolism
pelo	unknown	*CG4168*	Transmission of nerve impulse	*Lac*	Cell adhesion
psq	Transcrition factor activity	*CG4500*	-	*mdy*	Regulation of nurse cells apoptosis
Rac1	Dendrite morphogenesis	*CG4527*	Serine/threonine kinase	*mei-S332*	Cell cycle
tollo	Serine/threonine kinase	*CG4707*	transcription regulator activity	*spin*	Programmed cell death
TfIIs	Transcription factor activity	*CG3704*	ATP binding	*Tor*	Insulin pathway/ growth
		CG4973	Protein ubiquitination	*unk*	Larval development fate commitment

We found 86 genes that are highly correlated (coeff> 0,9). Among the 86 genes, 28 were also found to be overexpressed in the *V1* AMC (column on the left, gene names are indicated in bold) and contain the common putative *pros* DNA motif in their promoter. The 58 genes present in the two columns on the right are specifically overexpressed in *V1* CNS as compared to *V14* CNS.

Table S2: Phenotype data related to the candidate genes involved in neurite outgrowth and/or synaptic transmission.

Genes	**Tissue/organ/development stages**	**Phenotypes**	**Ref**
αEst1	Microarray data on adult and embryo	Putative target of pumillo that involved in morphogenesis of larval peripheral sensory neurons.	1
Ash2	Third instar larval brain<br>Larval motoneurons	Ash2 mutant present fasciculation defects in the ventral ganglion.<br>Ash2 activation generates a reduced synapses and pathfinding defects.	2<br>3
Bnl	Larval and adult Dorsal Cluster Neurons (DCNs).	Axon retraction.	6
CaMKI	Various tissue for larvae , pupae and adult	A Ca2+ sensor for a broad diversity of retinal proteins, some of which are implicated in synaptic transmission.	8
CG6388	larval Neuromuscular Junction	Overexpression of CG6388 suppresses the NMJ overgrowth and restaure the normal morphology	11
DPAL1	Embryos, larvae and adult.	Associated with specific peptidergic neurons. Involved in neuropeptide biosynthesis.	13
EGFR	Larva/pupal/adult ocellar sensory neuron.	Expression of a dominant negative form of EGFR resulted in stalling of axon extension in ocellar sensory neurons.<br>Axon sorting and extension	14
Gwl	Larval neuromuscular system (ISN)<br>Larval brain	GOF phenotype: ISNb (intersegmental neurons) pathfinding defects, including ectopic synaptic branches.<br>Larval brain neuroblasts are mitotic progression defective	3<br>21
LimK1	Adult antennal lobe<br>Larval Neuromuscular jonction	Increase in Limk leads to petit synapses and to ectopic glomeruli<br>Inhibit axons growth	50<br>26
loco	Embryos	defects in ensheathment of longitudinal axon tracts	29
Nej	Larval neuromuscular synapses	The presynaptic overexpression of CREB impaired neurotransmitter release<br>Overexpression of Nej can act to inhibit presynaptic functional development	31<br>11
Notch	L3/pupal/Adult brain DC neurons.<br>Adult.<br>Larval/adult olfactory receptor neurons (ORNs).	Overexpression results in inhibition of axonal branching, a complete failure of innervation of the medulla and defects in the arborisation pattern.<br>Overexpression produces an improvement in **olfactory** long-term memory<br>Odorant receptor expression and the axonal targeting of ORNs were specified according to their Notch-mediated identities	32<br>33<br>35
Prosα7	Tird instar larva NMJ<br>Tird instar larva Mushroom body gamma neurons	Inhibition of the proteasome causes a rapid strengthening of neurotransmission.<br>Neuronal remodelling: involved in axon pruning (microarray data),	40<br>30
Prosβ2	Third instar larval (NMJ)	Constitutive expression of prosβ2 induce an increase of excitatory junctional current (EJC) amplitudes.<br>Neuronal remodelling: involved in axon pruning (microarray data),	38<br>30

Table S2 continued

Prosα6	Tird instar larva NMJ	Inhibition of the proteasome causes a rapid strengthening of neurotransmission.<br>Neuronal remodelling: involved in axon pruning (microarray data),	40<br>30
Prosα26	Tird instar larva NMJ	Inhibition of the proteasome causes a rapid strengthening of neurotransmission.<br>Neuronal remodelling: involved in axon pruning (microarray data),	40<br>30
Pvr	CNS embryos	Required in midline glia (MG) during axon guidance and ultimately enabling axonal enwrapment. Required for axonal commissures to separate normally.<br>Removing the function of Pvr, or disrupting Rac1 function, inhibits VNC condensation<br>Pvr mutants have defects in axon scaffold formation in the CNS	44<br>51<br>52
Rac1	larval Dorsal Cluster Neurons (DCNs).<br>Larval Neuromuscular jonction<br>Larval PNS (da neurons)<br>Larvae « da » neurons	Activation of Rac1 inhibits axon extension and induces a decreased number of DCN axons crossing the optic chiasm.<br>Rac/Cdc42, (via effector kinases Rok or Pak), activate LIM kinase to inhibit axon growth<br>Elevated Rac1 causes increases in dentritic branching and filopodia formation in all da neurons<br>Overexpression of Rac1 promoted dendritic branching of "da" neurons	6<br>26<br>45<br>46
RPN1	Tird instar larva Mushroom body gamma neurons	Neuronal remodelling: involved in axon pruning (microarray data),	30
RPN2	Tird instar larva Mushroom body gamma neurons	Neuronal remodelling: involved in axon pruning (microarray data),	30
RPN5	Tird instar larva Mushroom body gamma neurons	Neuronal remodelling: involved in axon pruning (microarray data),	30
Tollo	Embryos/Larval	Heterochronic misexpression of Toll affects synaptogenesis and motoneuron growth cones	48

The criteria used for the description of the phenotypes were as follows: (1) if many larval phenotypes were available for a gene, we selected only those observed in the nervous system and preferentially in the peripheral nervous system (PNS). (2) If no larval phenotype was available for a gene, we selected those observed in the embryonic and/or adult PNS. (3) If available, the effect of the upregulation or downregulation of these genes is mentioned respectively for those that are overexpressed or underexpressed in *V1* AMC. (4) All studies mentioned were done in *Drosophila melanogaster*.

Table S3: Phenotype data related to the candidate genes involved in growth and autophagy.

Genes	**Tissue/organ/development stages**	Phenotyes	**Ref**
Ash2	Drosophila S2 cells<br><br>Third instar larvae	Resulted in an increased mean cell diameter when silenced<br>Increase of *ash2* protein was observed in mTOR knockdown cells.<br>Genetic mosaics, homozygous mutant cells show effects on both cell differentiation and cell size.	4<br><br>2
CG10702	Microarray data<br>Larval salivary gland	Insulin-like growth factor receptor activity: up-regulated by increases in EGFR and downregulated by loss of *EGFR.*<br>Autophagy	9<br>10
Dok	Embryo/adult	Insulin receptor binding, regulation of cell shape	12
EGFR	Embryos/Larval fat body<br><br>Microarray data	Impeding EGF signaling decreases cell size; Spi/EGFR activity promotes cell survival and growth but acts at a level that limits both processes.<br>Involved in autophagy in larval fat body (via keren ligand); insulin/TOR pathway regulates Neurogenesis and EGFR	15<br><br>16, 42
FK506-bp1	Fat body of feeding/wandering flies<br>Microarray data ; eye imaginal disc	Effect on autophagy, potentially through modulation of the transcription factor Foxo.<br>Overexpression inhibits cell growth, developmental and starvation-induced autophagy.	16
Ftz-F1	Salivary gland during developpement	Premature expression of *FTZ-F1* in larvae causes defects in the molting process.<br>Mutations in the steroid-regulated gene *FTZ-F1* prevents authophagic programmed cell death in salivary gland.	19
Iap2	larval salivary gland	Involved in autophagic cell death (identified by SAGE approach)	23
Keren	Larval fat body (microarray data)	Upregulated during developmental autophagy in larval fat body	16
LK6	Larvae<br><br>Adult	Overexpression of Lk6 results in growth inhibition in an eIF4E-dependent manner.<br>Regulation of growth<br>Lk6, involve in the TOR/FOXO nutrient sensing pathway.	28<br>27<br>53
Nej	Larval salivary gland	Autophagy (identified by micrroarray ananlysis)	10
Notch	Embryo (ovaries)<br>Third instar larvae	Interact with LK6 which involve in the TOR/FOXO pathway (growth and autophagy)<br>Notch coordinates in wing primordia tissue growth	20<br>49
PhKγ	Larvae	Glucose catabolic process	36
Pros β2	larval salivary gland	Autophagy (identified by micrroarray ananlysis)	10
Pros α7	larval salivary gland	Autophagy (identified by micrroarray ananlysis)	10
Prosα6	larval salivary gland	Autophagy (identified by micrroarray ananlysis)	10
Pros26	larval salivary gland	Autophagy (identified by micrroarray ananlysis)	10
Pros26.4	larval salivary gland	Autophagy (identified by micrroarray ananlysis)	10
RPN1	larval salivary gland	Autophagy (identified by micrroarray ananlysis)	10
RPN2	larval salivary gland	Autophagy (identified by micrroarray ananlysis)	10
RPN5	larval salivary gland	Autophagy (identified by micrroarray ananlysis)	10

The criteria used for the description of the phenotypes were the same as those for Table S2.

Table S4: Phenotype data related to the candidate genes involved in sensory organ development and most particularly in olfaction (in bold) .

Genes	**Tissue/organ/development stages**	**phenotypes**	**Ref**
Ash2	Wing imaginal disc	Preserve proper sensory organ organization. loss of ***ash2*** can yield ectopic sensory organs	5
CaMK1		Synaptic transmission	8
ckIIα	Adult eye	Compromising CK2 elicits surpernumerary R8 photoreceptor, rough eye and defects in the interommatidial bristles.	37
Dok	Adult	Loss of bristles in the midline region of the thorax, unilateral or bilateral loss of anterior orbital bristles in the head region and irregularly placed bristles in the eyes.	12
EGFR	Larva/pupae/adult ocellar sensory neuron<br>Pupae	Ocellar sensory neurons<br>Regulates cell number in the third segment of the antennae	14, 56<br>25
Ftz-F1	Adult abdominal and sternopleural bristles phenotypes	Loss of bristles<br>Mutants show an abnormal **olfactory** avoidance response.	54<br>41
Hb	embryos	Labial segment formation including sense organ	22
Iap2	Third instar larvae (wing disc)/adult notum	IAP2 overexpression results in additional macrochaetes<br>Transcript level increases in GOF EGFR mutant	24<br>9
Keren	Adult eye	Participate in EGFR signalling in the eye, where it acts redundantly with Spitz to control R8 spacing, cell clustering and survival	56
LimK1	Adult antennal lobe	Increase in Limk leads to petit synapses<br>presynaptic increase in Limk function leads to **ectopic glomeruli**	50
Mcr	Adult	P insertion in this gene induce **olfactory avoidance** behavior	17
Nak	Embryos chordotonal organ lineage	Overexpression of Nak causes both daughters of a normally asymmetric cell division to adopt the same cell fate and induces fate transformation from neuron to sheath cell.	18
Nej	P element insertion (3 kb up) effects on abdominal and sternopleural bristles	Gain of bristles	54
Notch	Pupae/adult antennae<br>Second instar larvae (imaginal disc)	O**lfactory sense-organs** : High Notch signaling and the exclusion of seven up, pros and elav markers identifies PIIa; this cell gives rise to the shaft and socket. See also Table 5<br>Growth promoted by Notch promotes growth in eye-antenna	34<br>7
pelo	Adult eye	*Pelota* mutant affects patterning in the eye. The eye is substantially smaller, with rough and disordered ommatidia and bristles.	55
Pros β2	Early pupal stage mechanosensory bristles	Decreased proteasome activity resulted inshaft-to-socket cell fate transformations and enhance Notch signaling activity in the sense organ lineage.	39

Table S4 continued

psq	Embryon/Larvae/ adult<br>P element insertions effects on abdominal and sternopleural bristles	Identification of psq mutant that shows an **olfactory** avoidance response<br>Loss of bristles	41<br>54
Pvr	Embryo/Larvae/adult	Macrochaete formation	43
Rac1	Embryos/larvae	Rac1 gain-of-function and loss-of-function mutants had both disruption of glial cell development and secondary effects on sensory axon fasciculation	47
tollo	Adult (P element insertions 5 kb up)<br>Indirect evidence	Gain of bristles<br>CD36 a co-factor of Tollo is expressed in a population of **olfactory** implicated in pheromone dedection	54<br>57

The criteria used for the description of the phenotypes were the same as those for Table S2.

REFERENCES

1. Gerber AP, Luschnig S, Krasnow MA, Brown PO, Herschlag D. Genome-wide identification of mRNAs associated with the translational regulator PUMILIO in Drosophila melanogaster. Proc Natl Acad Sci U S A 2006;103(12):4487-92.
2. Beltran S, Blanco E, Serras F, Perez-Villamil B, Guigo R, Artavanis-Tsakonas S, Corominas M. Transcriptional network controlled by the trithorax-group gene ash2 in Drosophila melanogaster. Proc Natl Acad Sci U S A 2003;100(6):3293-8.
3. Kraut R, Menon K, Zinn K. A gain-of-function screen for genes controlling motor axon guidance and synaptogenesis in Drosophila. Curr Biol 2001;11(6):417-30.
4. Guertin DA, Guntur KV, Bell GW, Thoreen CC, Sabatini DM. Functional genomics identifies TOR-regulated genes that control growth and division. Curr Biol 2006;16(10):958-70.
5. Adamson AL, Shearn A. Molecular genetic analysis of Drosophila ash2, a member of the trithorax group required for imaginal disc pattern formation. Genetics 1996;144(2):621-33.
6. Srahna M, Leyssen M, Choi CM, Fradkin LG, Noordermeer JN, Hassan BA. A signaling network for patterning of neuronal connectivity in the Drosophila brain. PLoS Biol 2006;4(11):e348.
7. Kenyon KL, Ranade SS, Curtiss J, Mlodzik M, Pignoni F. Coordinating proliferation and tissue specification to promote regional identity in the Drosophila head. Dev. Cell 2003;5(3):403-14.
8. Xu XZ, Wes PD, Chen H, Li HS, Yu M, Morgan S, Liu Y, Montell C. Retinal targets for calmodulin include proteins implicated in synaptic transmission. J Biol Chem 1998;273(47):31297-307.
9. Jordan KC, Hatfield SD, Tworoger M, Ward EJ, Fischer KA, Bowers S, Ruohola-Bake rH. Genome wide analysis of transcript levels after perturbation of the EGFR pathway in the Drosophila ovary. Dev. Dyn. 2005;232(3):709-24.
10. Martin DN, Balgley B, Dutta S, Chen J, Rudnick P, Cranford J, Kantartzis S, DeVoe DL, Lee C, Baehrecke EH. Proteomic analysis of steroid-triggered autophagic programmed cell death during Drosophila development. Cell Death Differ. 2007;14(5):916-23.
11. Laviolette MJ, Nunes P, Peyre JB, Aigaki T, Stewart BA. A genetic screen for suppressors of Drosophila NSF2 neuromuscular junction overgrowth. Genetics 2005;170(2):779-92.
12. Biswas R, Stein D, Stanley ER. Drosophila Dok is required for embryonic dorsal closure. Development 2006;133(2):217-27.
13. Han M, Park D, Vanderzalm PJ, Mains RE, Eipper BA, Taghert PH. Drosophila uses two distinct neuropeptide amidating enzymes, dPAL1 and dPAL2. J Neurochem 2004;90(1):129-41.
14. Garcia-Alonso L, Romani S, Jimenez F. The EGF and FGF receptors mediate neuroglian function to control growth cone decisions during sensory axon guidance in Drosophila. Neuron 2000;28(3):741-52.
15. Parker J. Control of compartment size by an EGF ligand from neighboring cells. Curr Biol 2006;16(20):2058-65.
16. Juhasz G, Puskas LG, Komonyi O, Erdi B, Maroy P, Neufeld TP, Sass M. Gene expression profiling identifies FKBP39 as an inhibitor of autophagy in larval Drosophila fat body. Cell Death Differ 2007;14(6):1181-90.
17. Anholt RR, Mackay TF. The genetic architecture of odor-guided behavior in Drosophila melanogaster. Behav Genet 2001;31(1):17-27.

18. Chien CT, Wang S, Rothenberg M, Jan LY, Jan YN. Numb-associated kinase interacts with the phosphotyrosine binding domain of Numb and antagonizes the function of Numb in vivo. Mol Cell Biol 1998;18(1):598-607.
19. Yamada M, Murata T, Hirose S, Lavorgna G, Suzuki E, Ueda H. Temporally restricted expression of transcription factor betaFTZ-F1: significance for embryogenesis, molting and metamorphosis in Drosophila melanogaster. Development 2000;127(23):5083-92.
20. Yan N, Macdonald PM. Genetic interactions of Drosophila melanogaster arrest reveal roles for translational repressor Bruno in accumulation of Gurken and activity of Delta. Genetics 2004;168(3):1433-42.
21. Yu J, Fleming SL, Williams B, Williams EV, Li Z, Somma P, Rieder CL, Goldberg ML. Greatwall kinase: a nuclear protein required for proper chromosome condensation and mitotic progression in Drosophila. J Cell Biol. 2004;164(4):487-92.
22. Wu X, Vasisht V, Kosman D, Reinitz J, Small S. Thoracic patterning by the Drosophila gap gene hunchback. Dev. Biol. 2001;7(1):79-92.
23. Gorski SM, Chittaranjan S, Pleasance ED, Freeman JD, Anderson CL, Varhol RJ, Coughlin SM, Zuyderduyn SD, Jones SJ, Marra MA. A SAGE approach to discovery of genes involved in autophagic cell death. Curr Biol 2003;13(4):358-63.
24. Kanuka H, Kuranaga E, Takemoto K, Hiratou T, Okano H, Miura M. Drosophila caspase transduces Shaggy/GSK-3beta kinase activity in neural precursor development. EMBO J. 2005;24(21):3793-806.
25. Sen A, Shetty C, Jhaveri D, Rodrigues V. Distinct types of glial cells populate the Drosophila antenna. BMC Dev Biol 2005;5:25.
26. Ng J, Luo L. Rho GTPases regulate axon growth through convergent and divergent signaling pathways. Neuron 2004;44(5):779-93.
27. Arquier N, Bourouis M, Colombani J, Léopold P. Drosophila Lk6 kinase controls phosphorylation of eukaryotic translation initiation factor 4E and promotes normal growth and development. Curr. Biol. 2005;15(1):19-23.
28. Reiling JH, Doepfner KT, Hafen E, Stocker H. Diet-dependent effects of the Drosophila Mnk1/Mnk2 homolog Lk6 on growth via eIF4E. Curr Biol 2005;15(1):24-30.
29. Granderath S, Klambt C. Identification and functional analysis of the Drosophila gene loco. Methods Enzymol 2004;389:350-63.
30. Martin DN, Balgley B, Dutta S, Chen J, Rudnick P, Cranford J, Kantartzis S, DeVoe DL, Lee C, Baehrecke EH. Proteomic analysis of steroid-triggered autophagic programmed cell death during Drosophila development. Cell Death Differ. 2007;14(5):916-23.
31. Marek KW, Ng N, Fetter R, Smolik S, Goodman CS, Davis GW. A genetic analysis of synaptic development: pre- and postsynaptic dCBP control transmitter release at the Drosophila NMJ. Neuron 2000;25(3):537-47.
32. Hassan BA, Bermingham NA, He Y, Sun Y, Jan YN, Zoghbi HY, Bellen HJ. atonal regulates neurite arborization but does not act as a proneural gene in the Drosophila brain. Neuron 2000;25(3):549-61.
33. Ge X, Hannan F, Xie Z, Feng C, Tully T, Zhou H, Xie Z, Zhong Y. Notch signaling in Drosophila long-term memory formation. Proc Natl Acad Sci U S A. 2004;101(27):10172-6.
34. Sen A, Reddy GV, Rodrigues V. Combinatorial expression of Prospero, Seven-up, and Elav identifies progenitor cell types during sense-organ differentiation in the Drosophila antenna. Dev Biol 2003;254(1):79-92.
35. Endo K, Aoki T, Yoda Y, Kimura K, Hama C. Notch signal organizes the Drosophila olfactory circuitry by diversifying the sensory neuronal lineages. Nat Neurosci 2007;10(2):153-60.

36. Bahri SM, Yang X, Chia W. The Drosophila bifocal gene encodes a novel protein which colocalizes with actin and is necessary for photoreceptor morphogenesis. Mol Cell Biol 1997;17(9):5521-9.
37. Bose A, Kahali B, Zhang S, Lin JM, Allada R, Karandikar U, Bidwai AP. Drosophila CK2 regulates lateral-inhibition during eye and bristle development. Mech Dev 2006;123(9):649-64.
38. Haas KF, Miller SL, Friedman DB, Broadie K. The ubiquitin-proteasome system postsynaptically regulates glutamatergic synaptic function. Mol Cell Neurosci. 2007;35(1):64-75.
39. Schweisguth F, Posakony JW. Antagonistic activities of Suppressor of Hairless and Hairless control alternative cell fates in the Drosophila adult epidermis. Development 1994;120(6):1433-41.
40. Speese SD, Trotta N, Rodesch CK, Aravamudan B, Broadie K. The ubiquitin proteasome system acutely regulates presynaptic protein turnover and synaptic efficacy. Curr Biol 2003;13(11):899-910.
41. Sambandan D, Yamamoto A, Fanara JJ, Mackay TF, Anholt RR. Dynamic genetic interactions determine odor-guided behavior in Drosophila melanogaster. Genetics 2006;174(3):1349-63.
42. McNeill H, Craig GM, Bateman JM. Regulation of neurogenesis and epidermal growth factor receptor signaling by the insulin receptor/target of rapamycin pathway in Drosophila. genetics 2008;179(2):843-53.
43. Ishimaru S, Ueda R, Hinohara Y, Ohtani M, Hanafusa H. PVR plays a critical role via JNK activation in thorax closure during Drosophila metamorphosis. EMBO J. 2004;23(20):3984-3994.
44. Learte AR, Forero MG, Hidalgo A. Gliatrophic and gliatropic roles of PVF/PVR signaling during axon guidance. Glia 2008;56(2):164-76.
45. Andersen R, Li Y, Resseguie M, Brenman JE. Calcium/calmodulin-dependent protein kinase II alters structural plasticity and cytoskeletal dynamics in Drosophila. J Neurosci 2005;25(39):8878-88.
46. Lee A, Li W, Xu K, Bogert BA, Su K, Gao FB. Control of dendritic development by the Drosophila fragile X-related gene involves the small GTPase Rac1. Development 2003;130(22):5543-52.
47. Sepp KJ, Auld VJ. RhoA and Rac1 GTPases mediate the dynamic rearrangement of actin in peripheral glia. Development 2003;130(9):1825-35.
48. Rose D, Zhu X, Kose H, Hoang B, Cho J, Chiba A. Toll, a muscle cell surface molecule, locally inhibits synaptic initiation of the RP3 motoneuron growth cone in Drosophila. Development 1997;124(8):1561-71.
49. Rafel N, Milán M. Notch signalling coordinates tissue growth and wing fate specification in Drosophila. Development. 2008;135(24):3995-4001.
50. Ang LH, Chen W, Yao Y, Ozawa R, Tao E, Yonekura J, Uemura T, Keshishian H, Hing H. Lim kinase regulates the development of olfactory and neuromuscular synapses. Dev Biol 2006;293:178-90.
51. Olofsson B, Page DT. Condensation of the central nervous system in embryonic Drosophila is inhibited by blocking hemocyte migration or neural activity. Dev Biol 2005;279(1):233-43.
52. Sears HC, Kennedy CJ, Garrity PA. Macrophage-mediated corpse engulfment is required for normal Drosophila CNS morphogenesis. Development 2003;130(15):3557-65.

53. Teleman AA, Hietakangas V, Sayadian AC, Cohen SM. Nutritional control of protein biosynthetic capacity by insulin via Myc in Drosophila. Cell Metab 2008;7(1):21-32.
54. Norga KK, Gurganus MC, Dilda CL, Yamamoto A, Lyman RF, Patel PH, Rubin GM, Hoskins RA, Mackay TF, Bellen HJ. Quantitative analysis of bristle number in Drosophila mutants identifies genes involved in neural development. Curr Biol 2003;13(16):1388-96.
55. Eberhart CG, Wasserman SA. The pelota locus encodes a protein required for meiotic cell division: an analysis of G2/M arrest in Drosophila spermatogenesis. Development 1995;121(10):3477-86.
56. Brown KE, Freeman M. Egfr signalling defines a protective function for ommatidial orientation in the Drosophila eye. Development 2003;130(22):5401-12.
57. Benton R, Vannice KS, Vosshall LB. An essential role for a CD36-related receptor in pheromone detection in Drosophila. Nature 2007;450(7167):289-93.
